# Supplementary material for: Implementing the reuse of public DIA proteomics datasets: from the PRIDE database to Expression Atlas
Source: Sci Data. 2022 Jun 14;9:335. doi: 10.1038/s41597-022-01380-9 (PMC9197839; doi:10.1038/s41597-022-01380-9)
Supplement: Supplementary file 1 — Supplementary Material [file 41597_2022_1380_MOESM1_ESM.pdf]

Supplementary Material

Table of contents:

Supplementary Table S1	p. 2
Supplementary Table S2	p. 3
Supplementary Table S3	p. 4
Supplementary Table S4	p. 5
Supplementary Figure S1	p. 6
Supplementary Figure S2	p. 7
Supplementary Figure S3	p. 8
Supplementary Figure S4	p. 9
Supplementary Figure S5	p. 10

Supplementary Table S1. Common protein accessions in significantly differentially expressed proteins in the original analysis and the reanalysis

PXD	Contrast	Protein Accessions
PXD000672	Normal vs ccRCC	P07148, P63104, P42126, P10809, P48047, Q86YH6, Q92597, P00740, P50238, P99999, P09110, Q9UHK6, P24539, Q02252, P43487, P22352, P08195, O75947, P04406, P45954, P47914, Q16718, Q16775, Q03154, O43175, Q9ULZ3, P40121, P24752, Q9H0W9, P18669, P25705, Q5T2W1, O75964, P00558, P15924, O95831, Q6PCB0, P07237, P09467, P01023, P30038, Q9H299, P20711, P06748, Q00325, P08758, P21912, P31937, P36543, P02792, P46777, P00966, P15880, P24821, Q13825, P06576, O75340, Q04941, P00918
PXD000672	ccRCC vs pRCC	Q99623, P00367, P35232, P55084, Q16891, P40939, P30044, Q9Y277, Q9UJZ1, P10253, Q13642, P53597, Q08380, P09110, Q13011, P10809, Q5VWZ2, P61604, P54819, P38117, P02794, P56385, O75390, O75964, Q16836, P31040, Q07021, Q9H2U2, P49748, P30084, P30049, P30048, Q16698, P36542, P40926, Q02252, Q9HCC0, P48047, P05091, Q00325, P12277, O43615, P06576, P05141, Q6IAA8, P11310, Q86SX6, Q99798, O95831, P25705, P36957, O75947, P49411, Q9Y2X3, P11177, P00403, Q9BX68, P10606, P13073, P99999, P42126, P18859, P24752, P24539, Q8NE62, P00505, P14406, Q9Y5L4, P45954, Q7Z4W1, Q13564, Q6P587, P08574, Q13423, P00846, P12235, P50440, P82933, O43169, Q9UHK6, Q969I3, P09669, O14880, Q13825, P12110, Q14315, P19827, P02656, Q15121, P10412, P00738, P50454, Q92597, P51884, P67936, O60701, P05155, P60981, P00338, P20962, Q15847, Q9H299, P02790, P01876, P30101, P35579, Q93088, P60660, P00558, P01024, O00299, P09525, P09493, P01834, P14618
PXD014943	eDLBCL vs PCNSL	P43403, P13760, Q15582, P14136, P04271, Q13885, P09543, P12277, Q9BVA1, P09471, P09936, Q12860, Q16555, P29992, Q12765, Q13449, P01871, Q8IXJ6, Q9UM22, P80723, P00568
PXD004691	Normal vs tumour (fresh frozen)	Q9BUD6, O95994, Q15063
PXD004691	Normal vs tumour (paraffin embedded)	P06748, P07585, P13647, P15309, P17661, P40926, Q04837, Q15063, Q8NBJ4

Supplementary Table S2. Quantified protein numbers per study using the 'top3' protein inference setting

Dataset	FDR	Reanalysis proteins	Original - before filter	Original - after filter	Reanalysis proteins ('50% missing)	Reanalysis proteins ('50% per group)	Peptides	Comment - original data
PXD004873	1%	3,530	N/A	2,579	3,530	3,392	22,231	0.1% FDR threshold
PXD000672	1%	4,053	N/A	1,632	4,053	4,037	31,888	0.1% FDR threshold
PXD004691	1%	2,872	N/A	3,030	2,872	2,686	17,943	No consistency filter mentioned
PXD014943	1%	5,946	N/A	5,769	5,946	5,568	45,986	No consistency filter mentioned
PXD003497	1%	2,754	6,873	3,700	2,752	2,704	20,296	6,873 filtered to 3,700 for subsequent analyses
PXD004589	1%	3,703	N/A	2,371	3,702	3,298	31,727	Number of proteins mentioned in supplementary material
PXD014194	1%	2,239	1,313	1,064	2,238	2,145	11,593	1313 filtered to 1064 for subsequent analyses
PXD003539	1%	7,097	6,556	3,171	7,096	6,867	77,014	Total number of proteins mentioned in supplementary material only (supplementary figure 2)
PXD001064	1%	207	425	342	207	197	3,508	425 on average, 342 selected for consistency
PXD010912	1%	4 ,224	N/A	1,250	4,224	4,123	34,776	Manuscript text provides protein number approximation and value range for individual runs

Supplementary Table S3. Quantified proteins per study using the 'all' protein inference setting

Dataset	FDR	Reanalysis proteins	Original - before filter	Original - after filter	Reanalysis proteins ('50% missing)	Reanalysis proteins ('50% per group)	Comment - original data
PXD004873	1%	3,530	N/A	2,579	2,925	2,297	0.1% FDR threshold
PXD000672	1%	4,053	N/A	1,632	3,932	2,648	0.1% FDR threshold
PXD004691	1%	2,872	N/A	3,030	2,033	1,688	No consistency filter mentioned
PXD014943	1%	5,946	N/A	5,769	4,336	3,879	No consistency filter mentioned
PXD003497	1%	2,754	6,873	3,700	2,576	2,109	6,873 filtered to 3,700 for subsequent analyses
PXD004589	1%	3,703	N/A	2,371	2,043	2,366	Number of proteins mentioned in supplementary material
PXD014194	1%	2,239	1,313	1,064	1,658	1,001	1,313 filtered to 1064 for subsequent analyses
PXD003539	1%	7,097	6,556	3,171	5,412	4,299	Total number of proteins mentioned in supplementary material only (supplementary figure 2)
PXD001064	1%	207	425	342	176	174	425 on average, 342 selected for consistency
PXD010912	1%	4,224	N/A	1,250	3,915	2,924	Manuscript text provides protein number approximation and value range for individual runs

Supplementary Table S4. Availability and location of intermediate results per study.

Dataset Identifier	.tsv TRIC output location
PXD004873	https://uk1s3.embassy.ebi.ac.uk/DIA-reanalysis/intermediate-results/tric_tsvs/PXD004873.tric.tsv.tar.gz
PXD000672	https://uk1s3.embassy.ebi.ac.uk/DIA-reanalysis/intermediate-results/tric_tsvs/PXD000672.tric.tsv.tar.gz
PXD004691	https://uk1s3.embassy.ebi.ac.uk/DIA-reanalysis/intermediate-results/tric_tsvs/PXD004691.tric.tsv.tar.gz
PXD014943	https://uk1s3.embassy.ebi.ac.uk/DIA-reanalysis/intermediate-results/tric_tsvs/PXD014943.tric.tsv.tar.gz
PXD003497	https://uk1s3.embassy.ebi.ac.uk/DIA-reanalysis/intermediate-results/tric_tsvs/PXD003497.tric.tsv.tar.gz
PXD004589	https://uk1s3.embassy.ebi.ac.uk/DIA-reanalysis/intermediate-results/tric_tsvs/PXD004589.tric.tsv.tar.gz
PXD014194	https://uk1s3.embassy.ebi.ac.uk/DIA-reanalysis/intermediate-results/tric_tsvs/PXD014194.tric.tsv.tar.gz
PXD003539	https://uk1s3.embassy.ebi.ac.uk/DIA-reanalysis/intermediate-results/tric_tsvs/PXD003539.tric.tsv.tar.gz
PXD001064	https://uk1s3.embassy.ebi.ac.uk/DIA-reanalysis/intermediate-results/tric_tsvs/PXD001064.tric.tsv.tar.gz
PXD010912	https://uk1s3.embassy.ebi.ac.uk/DIA-reanalysis/intermediate-results/tric_tsvs/PXD010912.tric.tsv.tar.gz

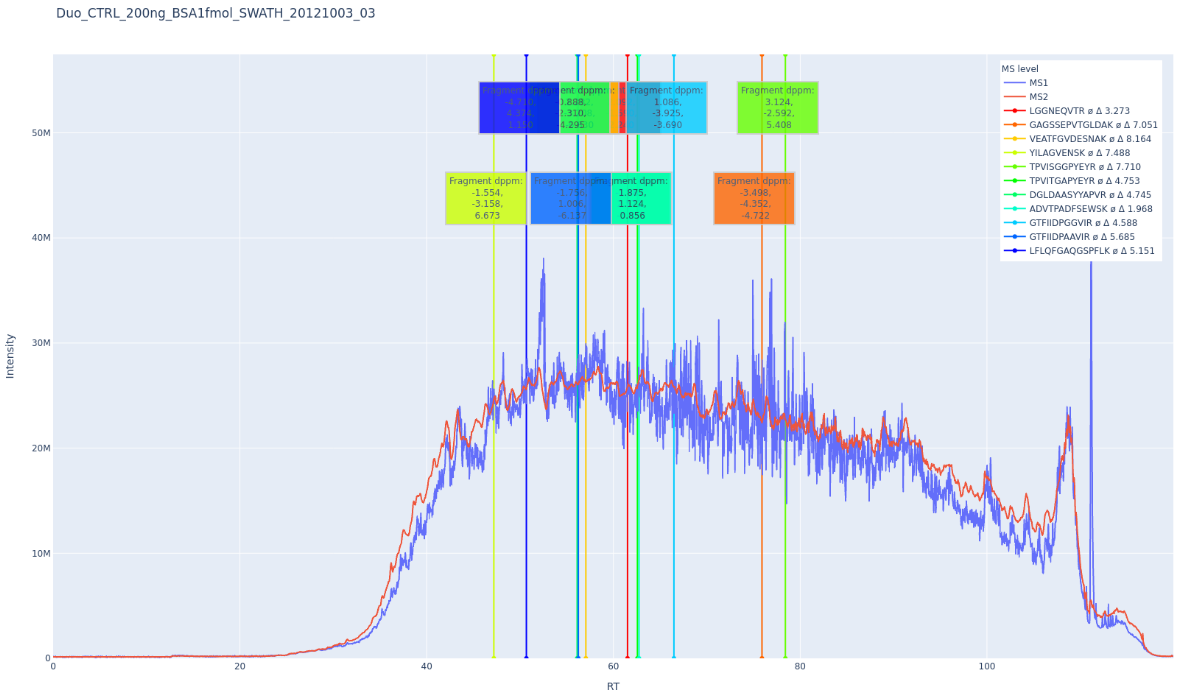

Supplementary Figure S1. QC plot of a SWATH-MS run from dataset PXD001506 with containing MS1 and MS2 TIC visualisation and labeled iRT peptide targets. A colour gradient from red to blue indicates the expected order of iRT peptides.

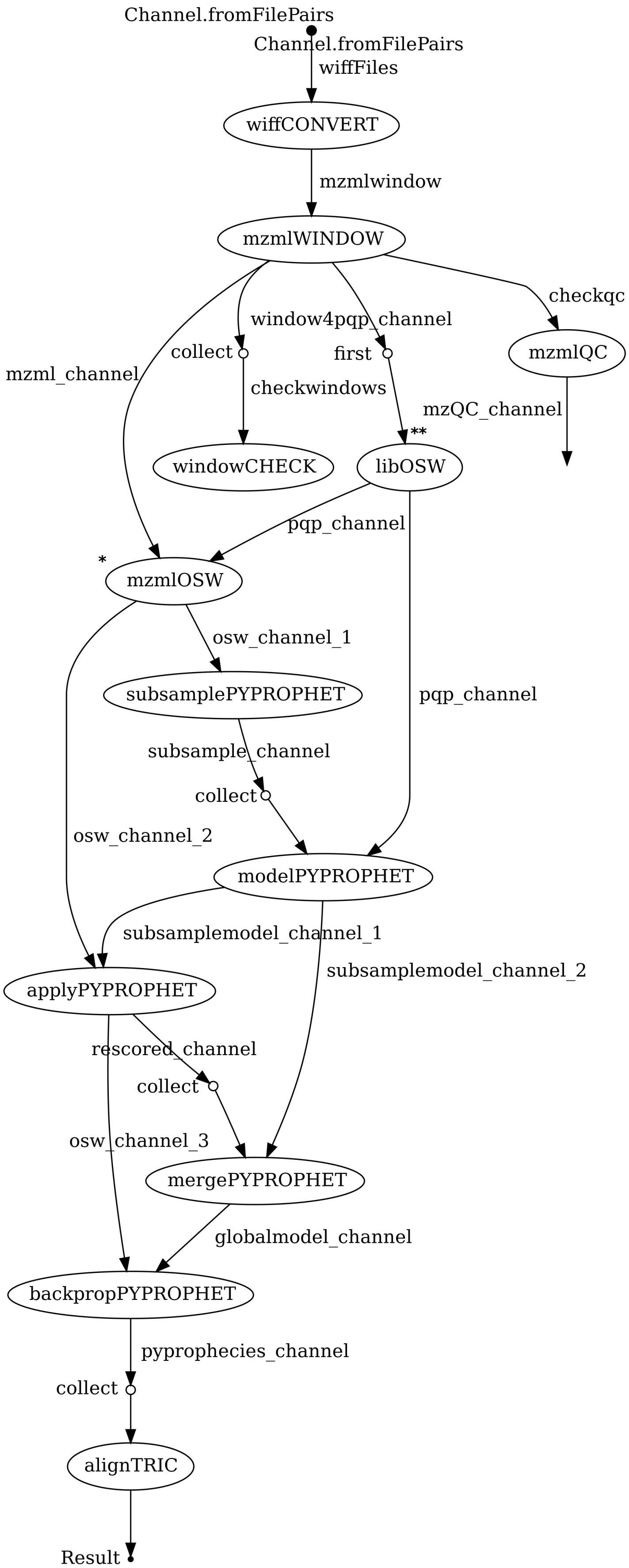

Supplementary Figure S2. Detailed visualisation of the upstream Nextflow workflow step sequences. Optional entry points with alternative inputs are marked with an asterisk.

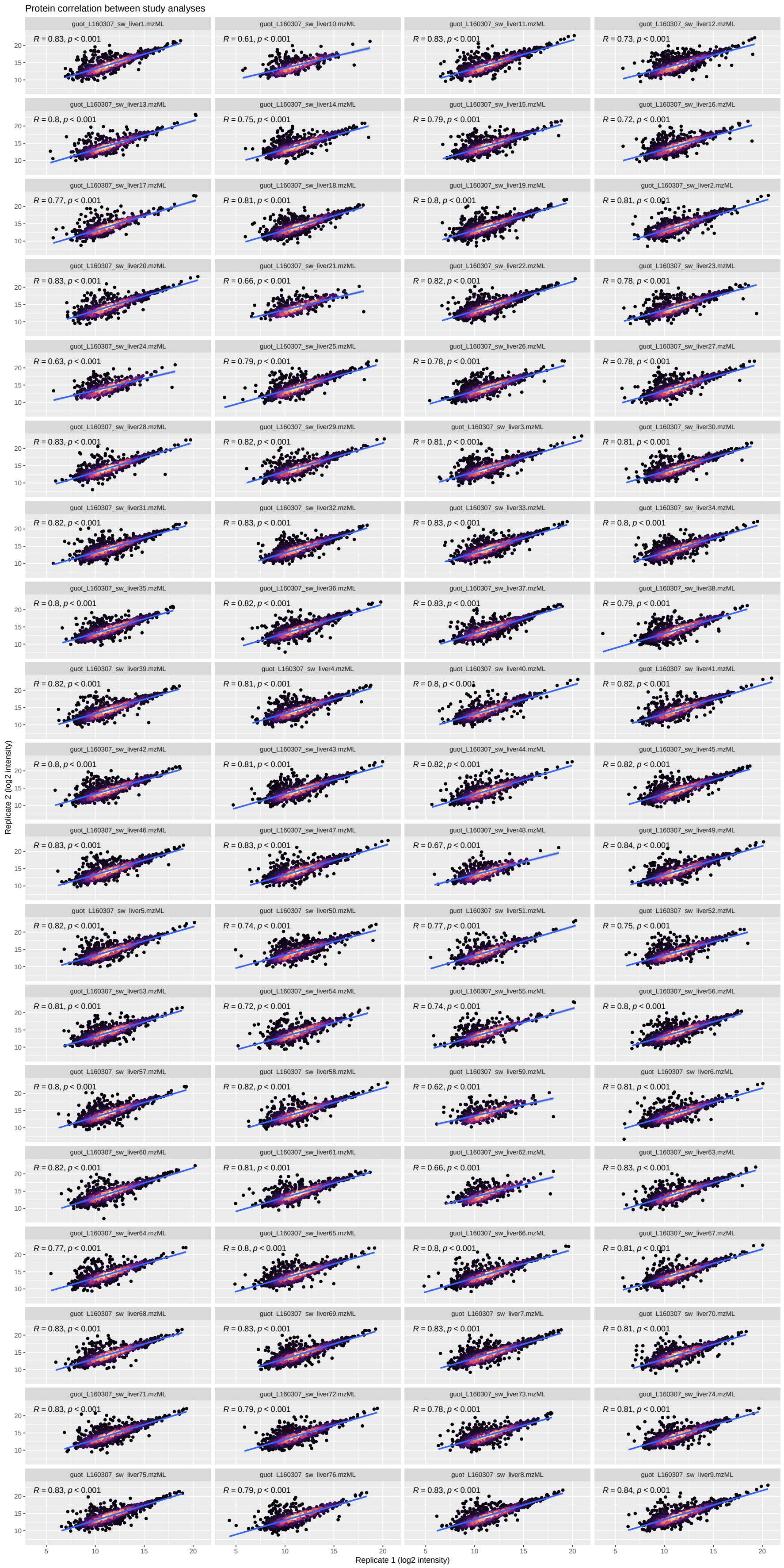

Supplementary Figure S3. Run-wise correlation of protein quantification results, reanalysed protein Intensities versus originally published protein intensities. Each one close-to-zero (< 0.05) intensity pair was removed from 5 runs (guot_L160307_sw_liver32.mzML, guot_L160307_sw_liver34.mzML, guot_L160307_sw_liver67.mzML, guot_L160307_sw_liver7.mzML, guot_L160307_sw_liver70.mzML).

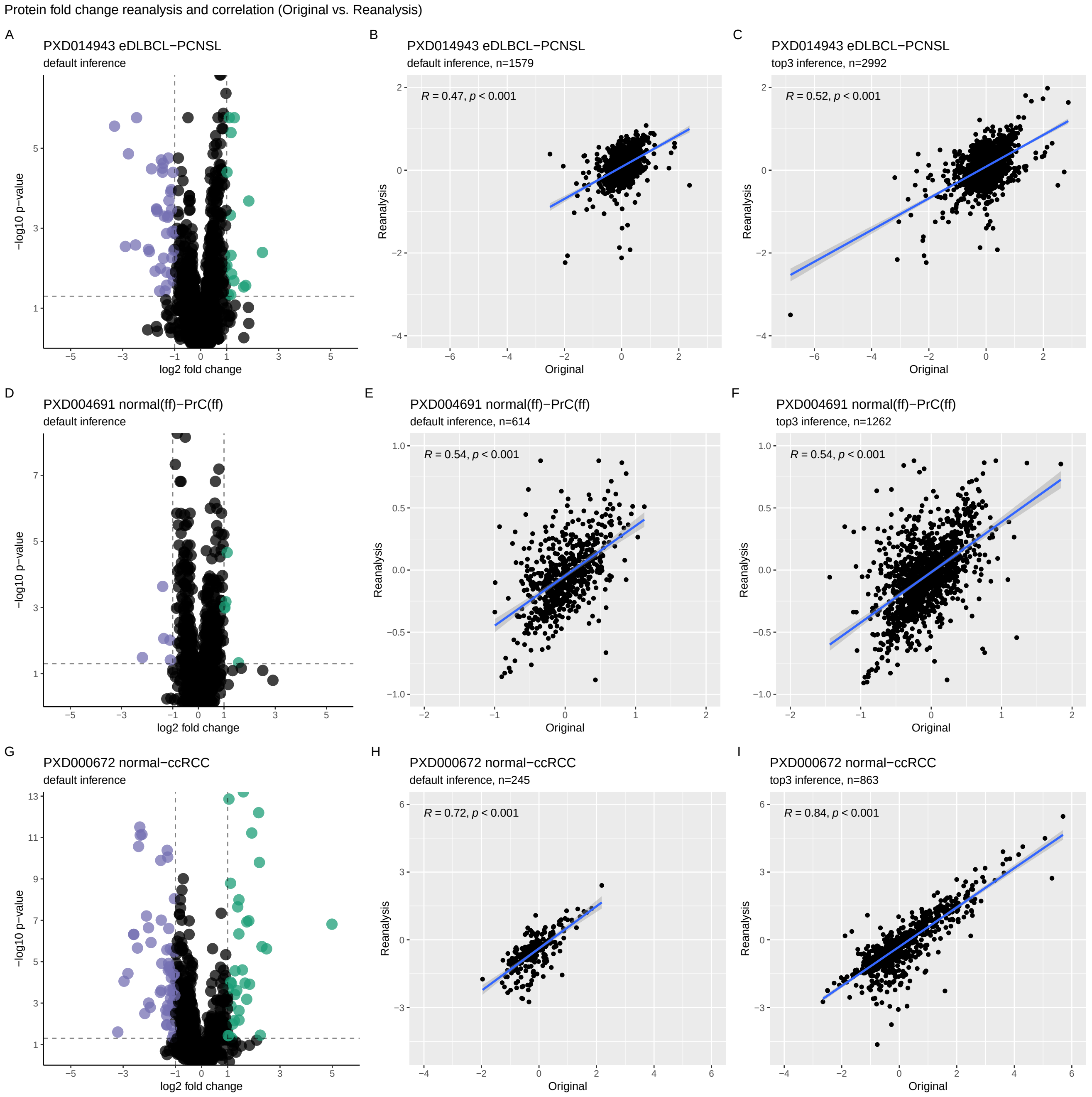

Supplementary Figure S4. Inference method influence on differential expression analysis. A, D, G) Volcano plots corresponding to 'all' protein inference method; B, E, H) Fold change (FC) log2 value correlations corresponding to 'all' protein inference method reanalysis vs original; C, F, I) FC log2 value correlations corresponding to 'top3' protein inference method reanalysis vs original. The FC compared are represented by points on the plot. Significant FC proteins are colour indicated. The dashed lines indicate the fold-change cutoff of 2 and the p-value cutoff at 0.05.

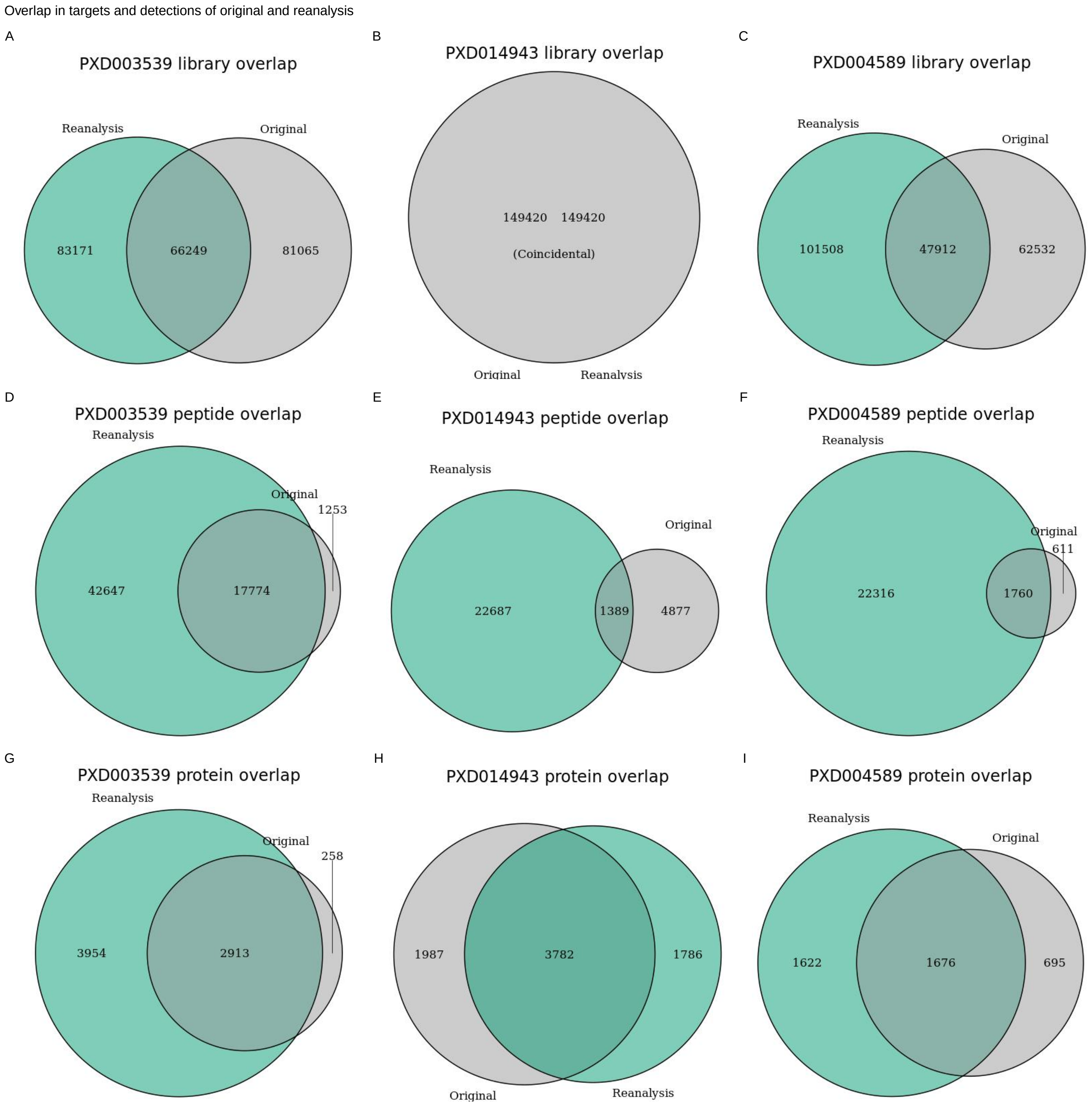

Supplementary Figure S5. Target library (A,B,C), peptide detection (D,E,F), and protein detection (G,H,I) overlaps for PXD003539, PXD014943, and PXD004589 between the originally published results and the reanalysis.
